# Epidemiology Profile of Viral Meningitis Infections Among Patients in Qatar (2015–2018)

**DOI:** 10.3389/fmed.2021.663694

**Published:** 2021-06-16

**Authors:** Shilu Mathew, Hebah A. Al Khatib, Khalid Al Ansari, Joanne Nader, Gheyath K. Nasrallah, Nadin N. Younes, Peter V. Coyle, Asmaa A. Al Thani, Muna A. Al Maslamani, Hadi M. Yassine

**Affiliations:** ^1^Biomedical Research Center, QU Health, Qatar University, Doha, Qatar; ^2^Emergency Department, Sidra Medicine, Doha, Qatar; ^3^Hamad Medical Corporation, Doha, Qatar; ^4^College of Health Sciences, QU Health, Qatar University, Doha, Qatar

**Keywords:** viral meningitis, epidemiology, enterovirus, genotyping, clinical outcome

## Abstract

**Background:** Little is known about the etiology of meningitis in the MENA region, including Qatar. Viral agents are considered the major cause for meningitis worldwide. Here, we present primary data about the etiology and clinical and demographic characteristics of viral meningitis (VM) in Qatar between 2015 and 2018.

**Methods:** We retrospectively collected data from Hamad Medical Corporation (HMC), which provides about 80% of healthcare services in Qatar. Data were collected for the period between 2015 and 2018. During this time period, 6,705 specimens were collected from patients with suspected meningitis attending HMC and primary healthcare centers. These specimens were tested for a panel of viruses using the “FTD Viral meningitis” multiplex real-time PCR kit that detects Adenovirus (ADV), Human herpesvirus 1&2 (HSV1 and HSV2), Epstein–Barr virus (EBV), Enteroviruses (EV), Cytomegalovirus (CMV), Varicella zoster virus (VZV), and Parechovirus (PV).

**Results:** Only 10.9% (732/6,705) of all suspected meningitis cases were caused by viral agents. 60.9% of the reported cases were males, compared to 39.1% in females. Most of the infections (73.9%) were reported in children younger than 10 years of age. EV were identified as the main causative agent (68.7%), followed by EBV (7.5%) and ADV (6.8%). Other viral agents including VZV, PV, HSV-1, and HSV-2 were also detected with a lower frequency. Confirmed VM were more prevalent among Qatari subjects compared to other nationalities. We observed no specific seasonality of viral agents, but a slight rise was recorded during the spring seasons (March to June). Fever (59.4%, 435/732) and acute central nervous system (CNS) infection (15.6%, 114/732) were initial symptoms of most cases.

**Conclusion:** This is the first report about the molecular epidemiology of VM in Qatar. In line with the international records, our data showed that EV is responsible for 68.7% of Qatar's VM cases. Further studies are needed to genotype and serotype the identified viruses.

## Introduction

Meningitis has a worldwide prevalence. The clinical and epidemiological characteristics are associated with infectious agents, socioeconomic characteristics of the population, and environmental factors. The infectious agents are majorly viruses and bacteria. Nevertheless, as the incidence of bacterial meningitis decreases, the proportion of meningitis cases caused by viruses is increasing (1). The burden of viral meningitis (VM) infection remains uncertain and poses a major public health challenge, specially in developing countries. VM is a serious health problem because of its progression in severity, the severe sequelae, and the potential risk of mortality (2). VM is commonly seen in children (<1-year old); however, it can affect other age groups as well (2). VM usually manifests with signs and symptoms that suggest its diagnoses, such as fever, headache, lethargy, irritability, loss of appetite, diarrhea, stiff neck, vomiting, anorexia, rash, meningeal irritation, and convulsions. However, these symptoms are general and can accompany other viral infections (3). Besides, in some cases, meningitis can progress into meningoencephalitis leading to seizures and mental disturbances (4).

Several common viruses can cause VM, including enteroviruses (EV) (5), herpes simplex viruses (HSV) (6), and mumps (7), which are considered the most important causative agents leading to VM. It is worth mentioning that EV is the cause of most VM cases (75–90%) in patients of all ages, in both endemic and epidemic patterns^1^. To be more specific, the agents that are grouped under EV from the Picornaviridae family, including coxsackieviruses (A1-22, 24, and B1-6), polioviruses (1–3), the enteroviruses 68, and echovirus, have been reported to be the most common viral agents causing the VM disease (8). On the other hand, rubella, cytomegalovirus (CMV), rabies virus, arboviruses, varicella-zoster virus (VZV), and measles are also associated with VM disease, but with a lower frequency (9).

VM outbreaks happen approximately every year; inpatient hospitalizations resulting from VM range from 25,000 to 50,000 each year in the United States^2^. In temperate climates, VM occurs monthly with a peak incidence of 1 per 100,000 persons^2^. However, many cases are undiagnosed because VM is a self-limited disorder that often resolves without symptoms^3^. Interestingly, the reported VM cases are the highest during the summer and early fall^2^.

VM is known to be a self-limited disease that could resolve independently without the need for treatment. Perhaps, patients with VM are known to have less severe consequences than bacterial meningitis; however, intravenous antiviral medication might be necessary in some extreme cases. Acyclovir is the most common intravenous treatment for cases infected with HSV and VZV. The treatment should be taken for 3 weeks, reducing mortality by 20% (10). Besides, Ganciclovir is the best choice for treating CMV infections, and it is supposed to be taken for 2 weeks (11). Recently, pleconaril has been clinically tested for EV infections. Although the drug showed effectiveness in many patients, concerns about drug interactions and side effects such as severe headache resulted in failure of license by the FDA (12). Further, studies have shown that intravenous immunoglobulin might be an alternative treatment for EV infections (13). Until the development of effective therapeutics, it is of prime importance to understand epidemiological and clinical patterns of viral meningitis, especially in developing countries, as this well help the public health authorities to implement the appropriate measures to control and manage the disease (14).

Very few studies have characterized population-based epidemiological distribution epidemiology and seasonality of VM viruses from the Middle East and North Africa (MENA). In Qatar, there is a rapidly growing diverse population due to the influx of foreign labor, where expatriates constitute about 87.3% of the total population (15). Thereby, this mixed population flow creates unique risks among healthcare, including the importation and spread of communicable diseases. So far, only data about the central nervous system (CNS) viral infections (16) and bacterial meningitis (17) have been reported in Qatar but not explicitly regarding VM epidemiology. Further detailed association between various VM agents with seasonality, clinical outcomes, and nationality has not been reported from any MENA region. Henceforth, we report a retrospective analysis utilizing the existing data from the VM surveillance system to explore the etiology, frequency, and pattern of circulation of VM infection in Qatar.

## Methods

### Sample Collection and Laboratory Screening

This is a retrospective study relying on data available at Hamad Medical Corporation (HMC) the major health provider in Qatar (>80% of the services). We retrieved all data related to CSF-test orders from the laboratory database for the period from 2015 to 2018. A total number of 6,705 patients were referred to the hospital due to meningitis-like symptoms. Patients ranged in age from 0 to 118 years. Patients with suspected viral meningitis-like symptoms (VMLS) were referred from Hamad General Hospital clinics and all primary healthcare centers (PHCC) in Doha between January 2015 and December 2018. This study was conducted in full accordance with the regulation of research at Hamad Medical Corporation (HMC) and Qatar University (QU): HMC-Institutional Review Board (HMC-IRB approval #17288/17) and QU-IRB (QU-IRB 998-E/18). Due to the lack of a standardized VMLS case definition, a strict diagnostic algorithm is still not available to detect different VM agents (zoonotic and non-zoonotic) (18). However, based on the physician's diagnosis, patients presenting with symptoms including acute onset of infection, inflammation, or rashes around the head, ears, throat, and the skin, followed by altered mental status or decreased level of consciousness or seizures or focal neurological signs, were considered VMLS. For viral detection, cerebral spinal fluid (CSF) samples were collected from patients at admission. Specimens were collected in a viral transport medium, stored at 4°C, and sent to the virology laboratory of HMC for testing.

Laboratory screening test for eight VM pathogens is routinely performed at the HMC virology lab using the FTD-viral meningitis diagnostic kit (Fast Track Diagnostics, Luxembourg). The kit is used for the detection of Epstein–Barr virus (EBV), Human herpesvirus 1&2 (HSV-1 and HSV-2), Varicella zoster virus (VZV), Cytomegalovirus (CMV), Enteroviruses (EV), Parechovirus (PV), and adenovirus (ADV). The limit of detection (LOD) of each assay was determined by analyzing serial dilutions of virus copy numbers ranging from 10^1^ to 10^6^ copies. The viral DNA or RNA copy numbers in clinical samples was quantified based on the threshold cycle (Ct) values of viral template calibrators.

### Statistical Analysis

Descriptive statistics were used to characterize the cohort of patients with VM infections. We classified the age of the patients into four main categories: children (<1–9 years of age), adolescents (10–17 years), adults (18–59), and elderly (>60 years). We then conducted a statistical analysis to analyze the demographic and clinical characteristics of subjects and to identify the frequency, patterns, and seasonality of all VM viruses. A Chi-square test for trend was used to evaluate the differences in VM infections over the years and among different age groups. Temporal trends in VM incidence rates over the 4 years of the study were assessed using the Extended Mantel–Haenszel chi-square test for linear trend using openepi tool^4^. *P* < 0.05 were considered statistically significant. All statistical analyses were done using GraphPad (Prism version 5.04) (IBM, Armonk, NY, USA).

## Results

### Patients Demographics

A total of 6705 specimens were collected at HMC from patients with VMLS between January 2015 and December 2018. Of those, 57.6% (3,866) of the samples belonged to males, while the rest were from females, 42.4% (2,839). This study included all age groups, with 67.8% of children (<1–9 years old) being the majority, followed by 25.1% adults (18–59 years old), 2.51% adolescents (10–17 years old), and 4.59% elderly (>60 years old) (Table 1). Out of the total tested specimens, 5,139 (76.6%) were collected from non-Qataris, whereas the remaining 1,566 (23.4%) were from Qataris. Distribution of subjects among non-Qatari populations included Indians (9.78%), Egyptians (8.46%), Pakistanis (8.47%), Sudanese (7.31%), Syrians (4.95%), Bangladeshis (4.62%), Nepalese (4.34%), Jordanian (3.18%), and Sri Lankans (1.81%). Many other nationalities were present but in lesser records (comprising 23.7%). Table 1 highlights the main demographic characteristics of the enrolled subjects.

**Table 1:** Demographic characteristics of the study population.

**Category**		**2015 No. (%)**	**2016 No. (%)**	**2017 No. (%)**	**2018 No. (%)**	**Total No. (%)**
**Gender**						
	Male	873 (60.9)	1,085 (59.7)	1,098 (52.1)	810 (60.0)	3,866 (57.6)
	Female	559 (39)	732 (40.3)	1,009 (47.9)	539 (40.0)	2,839 (42.4)
	Missing data	0 (0.0)	0 (0.0)	0 (0.0)	0 (0.0)	0 (0.0)
	Total	1,432 (100)	1,817 (100)	2,107 (100)	1,349 (100)	6,705 (100)
**Major nationalities**						
	Qatari	292 (20.3)	480 (26.4)	647 (30.8)	147 (11.0)	1,566 (23.1)
	Indian	138 (9.64)	232 (12.8)	223 (10.5)	63 (4.67)	656 (9.78)
	Egyptian	178 (12.4)	201 (11.0)	137 (6.50)	51 (3.78)	567 (8.46)
	Pakistani	172 (12.0)	166 (9.14)	170 (8.07)	60 (4.45)	568 (8.47)
	Syrian	96 (6.70)	79 (4.4)	88 (4.18)	69 (5.12)	332 (4.95)
	Jordanian	61 (4.26)	51 (2.8)	70 (3.32)	31(2.30)	213 (3.18)
	Sudanese	42 (2.93)	325 (17.8)	93 (4.41)	30 (2.22)	490 (7.31)
	Nepali	47 (3.28)	71 (4.0)	120 (5.70)	53 (3.92)	291 (4.34)
	Bangladesh	49 (3.42)	63 (3.4)	192 (9.11)	6 (0.44)	310 (4.62)
	Sri Lankans	31 (2.16)	41 (2.0)	27(1.28)	23 (1.70)	122 (1.81)
	Others	326 (22.8)	108(6.0)	340(16.1)	816 (60.5)	1,590 (23.7)
	Missing data	0 (0.0)	0 (0.0)	0 (0.0)	0 (0.0)	0 (0.0)
	Total	1,432 (100)	1,817 (100)	2,107 (100)	1349 (100)	6,705 (100)
**Age groups**						
	>1–9	896 (62.6)	1,320 (72.6)	1,432 (65.2)	901 (66.8)	4,549 (67.8)
	10–17	44 (3.07)	33 (1.82)	71 (3.37)	20 (1.48)	168 (2.51)
	18–59	398 (27.77)	401 (22.1)	500 (23.7)	381 (28.2)	1,680 (25.1)
	>60	94 (6.56)	63 (3.47)	104 (4.94)	47 (3.48)	308 (4.59)
	Total	1,432 (100)	1,817 (100)	2,107 (100)	1,349 (100)	6,705 (100)

### Circulating VM Viruses

Out of 6705 samples tested throughout the study period, 732 (10.9%) were considered positive for at least one VM agent. Table 2 summarizes the distribution of different VM pathogens by year of detection. Of the positive samples, EV was the most frequently detected virus, comprising 68.7% (503/732) of cases, followed by EBV (55/732, 7.5%), ADV (50/732, 6.8%), and CMV (33/732, 4.5%). Other VM agents were also reported with lower frequencies, including VZV (35/732, 4.8%), PV (30/732, 4.1%), HSV1 (12/732, 1.6%), and HSV2 (14/732, 1.9%).

**Table 2:** Distribution of different VM pathogens by year of detection.

	**Surveillance year**						
	**All years**	**2015**	**2016**	**2017**	**2018**		
	**Positive cases**						
**Pathogen**	**No. (%)**	**No. (%)**	**No. (%)**	**No. (%)**	**No. (%)**	***X^**2**^***	*p*–value^*^
ADV	50 (100)	13 (26)	18 (36)	12 (24)	7 (14)	3.43	*P >* 0.05
HSV1	12 (100)	1 (8.33)	3 (25)	4 (33.33)	4 (33.33)	3.69	*P >* 0.05
HSV2	14 (100)	2 (14.3)	3 (21.43)	8 (57.14)	1 (7.14)	0.01	*P >* 0.05
EBV	55 (100)	13 (23.64)	13 (23.64)	13 (23.64)	16 (29.1)	1.15	*P >* 0.05
EV	503 (100)	55 (10.93)	136 (27)	246 (48.9)	66 (13.12)	9.61	*P < * 0.001
CMV	33 (100)	4 (12.12)	13 (39.39)	11 (33.33)	5 (15.15)	0.02	*P >* 0.05
VZV	35 (100)	9 (25.71)	9 (25.71)	8 (22.86)	9 (25.71)	0.03	*P >* 0.05
PV	30 (100)	5 (16.67)	10 (33.33)	9 (30)	6 (20)	0.07	*P >* 0.05
Total	732	102	205	311	114		

* *P-values were obtained using Extended Mantel-Haenszel chi square test for linear trend*.

### Patterns of Circulation

**Figure 1:**
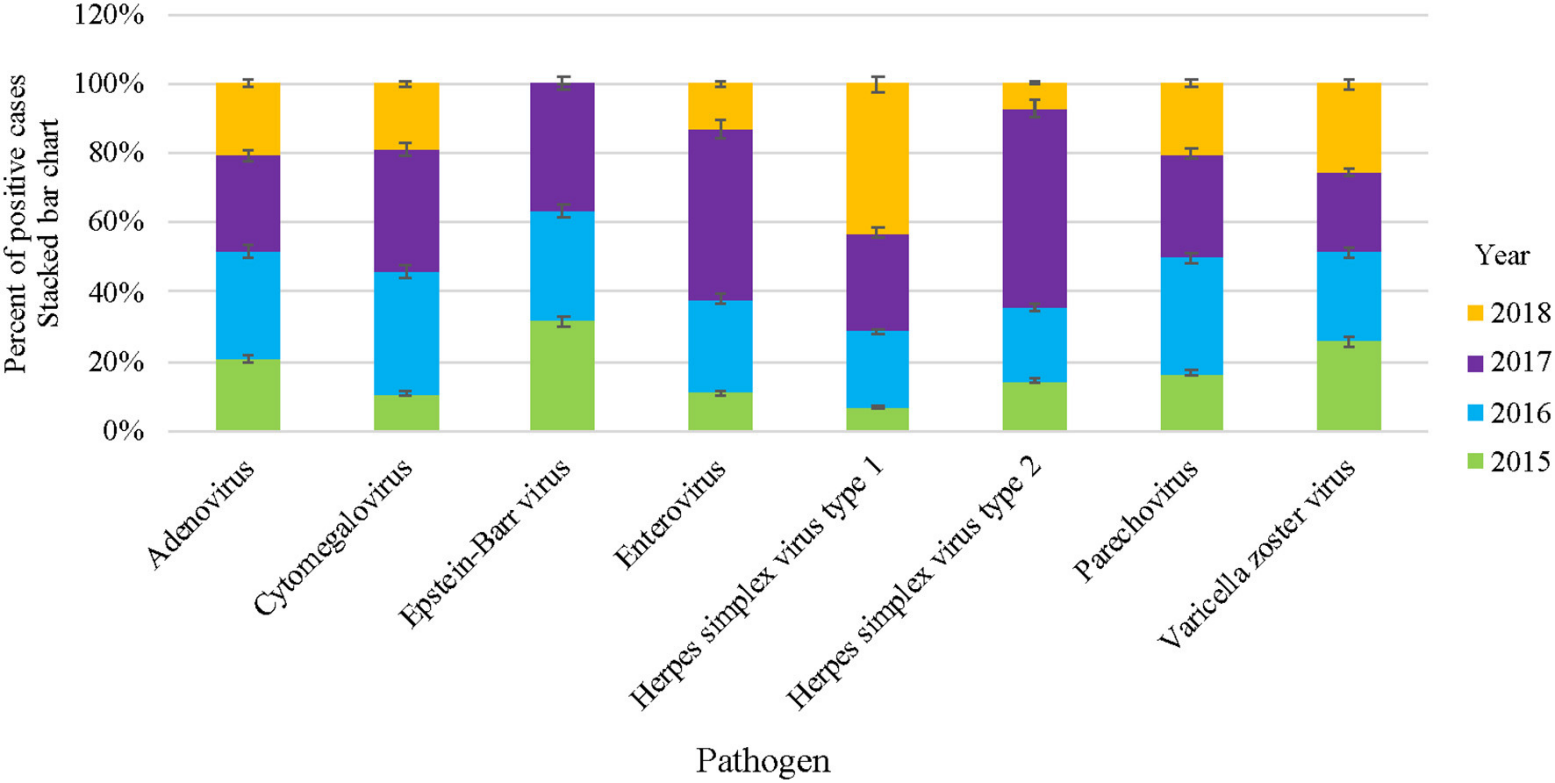
Overall VM cases reported in HMC during 2015–2018. Stacked bar chart denoting the percentage of VM positive cases of each specific virus, represented as Y-axis and pathogens as X-axis, respectively.

The circulation patterns of VM infections were almost similar throughout the study period. The number of VM-positive pathogens increased gradually during the first 3 years of the study (2015–2017), with samples ranging from 102 in 2015 (13.9%) to 311 (42.5%) samples in 2017 (Table 2). However, there was a drop in the positive samples in 2018 to 114 (15.6%) samples. In general, EV was recorded to be the most dominant VM in all 4 years of the study, with rates ranging from 53% in 2015 to 80% in 2017 (Figure 1). EBV and ADV were the second and third most frequently detected viruses, with a positivity rate of 5.7 and 6.8%, respectively (Table 2). EBV detection rate was 12.7% in 2015 and then decreased in 2016 (6.3%) and 2017 (4.2%). EBV detection rate was highest in 2018 with 14%. Similarly, the ADV was positively detected in 2015 (12.7%) and then gradually dropped in the following years to 6.1% in 2018. HSV1, PV, and CMV were detected higher between 2016 and 2018 compared to 2015 of all the VM positive samples (Table 2). Generally, the number of VM infections peaked in 2017, accounting for 42.5% (311/732) of the total positive samples.

### Patterns of Seasonality

**Figure 2:**
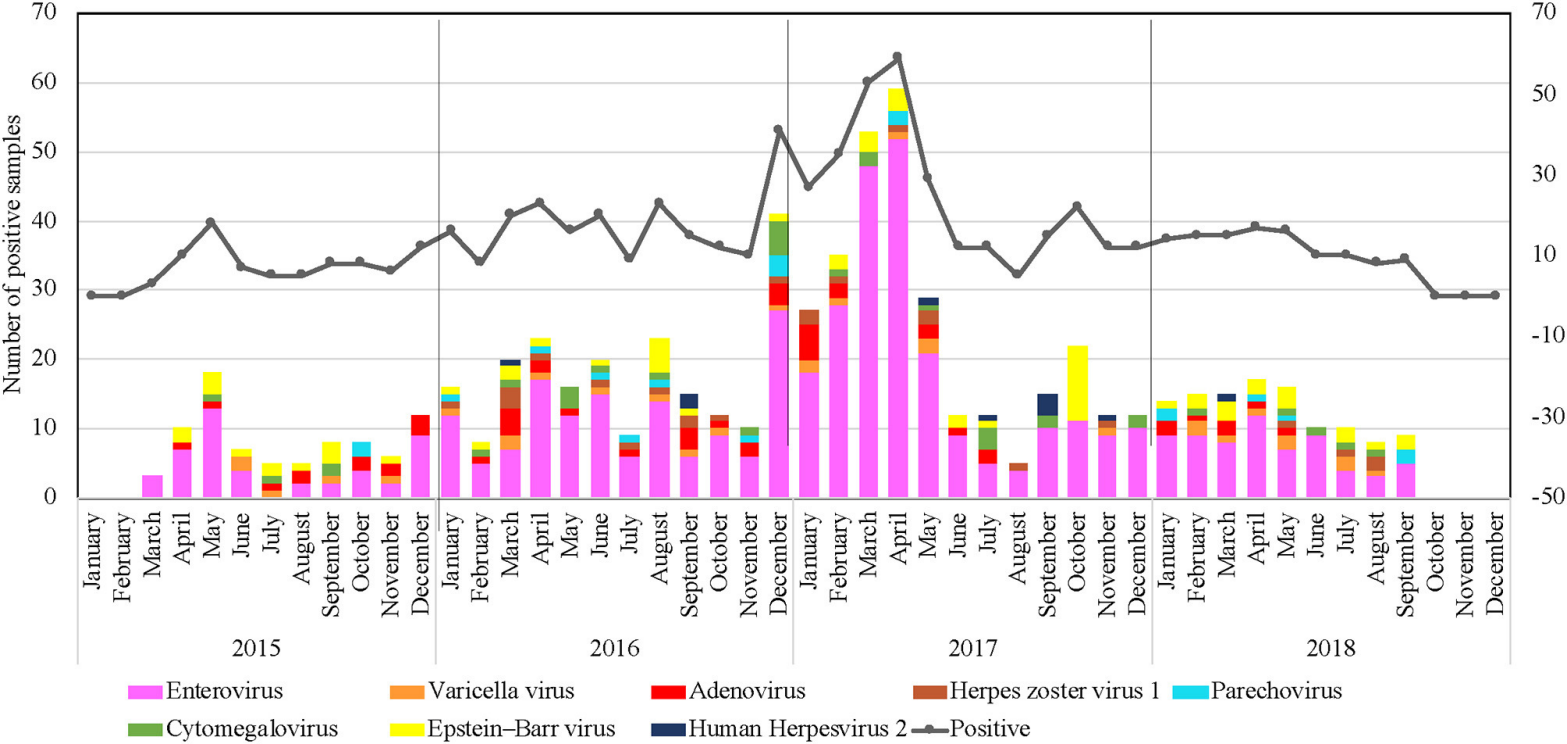
Clustered column chart denoting number of VM positive cases detected in Qatar during the study period (January 2015 to December 2018). X-axis: month; y-axis: Number of positive samples.

Qatar's climate is characterized by being dry, hot, and with low annual rainfall, especially in the summer season. Regardless of the climate in Qatar, our data indicated that VM infections circulated throughout the year. However, a significant circulation was identified during the spring seasons of all years (March to June), representing 47.8% of cases, as shown in Figure 2. On the other hand, a drop in the VM cases was recorded during winters (December to February) with 25.5%, followed by fall months between September and November (15.7%). Over the 4-years study period, the highest number of received samples was recorded during the period from December 2016 until May 2017 (35.8%) (Figure 2).

Consistently, EV circulated throughout the year; however, data from the 4 years identified significant peaks during the spring seasons (March–June). The highest detection rate was seen between December 2016 and May 2017, with a detection rate of up to 38.5% of all positive EV samples (Figure 2). Although VM infection's overall rate was considerably higher between the winter and spring seasons, the seasonality of different VM pathogens was variable, as illustrated in Figure 2. EBV ranked as the second-leading cause of VM infections that tends to circulate in a unique pattern with no detection in December. The highest detection rate was seen in October 2017, with a detection rate of up to 38.5% of all positive EBV samples. Virus seasonality was less interpretable for ADV, PV, HSV1, HSV2, and VZV since they were non-uniformly distributed all over the years, with no clear temporal patterns of infection fluctuations.

### Gender Distribution of VM Viruses

**Figure 3:**
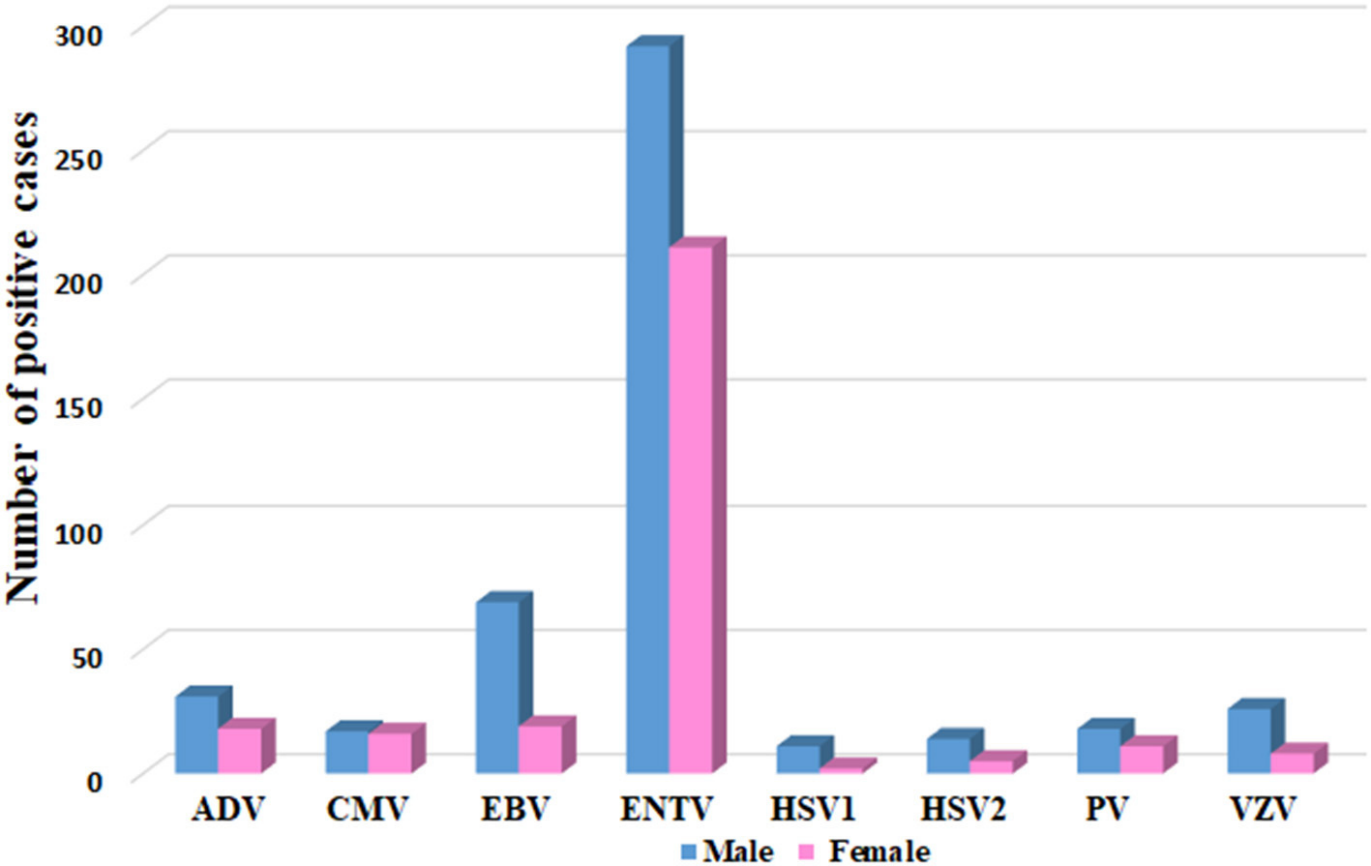
Gender distribution of VM agents, represented as number of positive cases in Y axis and each specific VM virus in Y-axis.

Most of the VM cases (60.9%; 446/732) were reported in males compared to 39.1% (286/732) in females. In general, males had a higher infection rate with all VM viral infections, as shown in Figure 3. Besides, throughout the 4 years of the study period, males had a higher infection rate with all VM viral infections, except in 2015, where the number of females infected with PV was higher than males (Supplementary Figure 1). Interestingly, in 2015, the positive cases for ADV, CMV, and HSV1 comprised only males. Likewise, positive cases for HSV1 were detected mainly among males in 2015, 2016, and 2018. Except in 2017, HSV1 was positive in both male and female cases (Supplementary Figure 1).

### Distribution of VM Cases in Different Age Groups

Among all the age groups, EV was the predominant infection, and it was more prevalent among children (85.1%, 428/503) compared to adolescents (8.94%, 45/503) and adults (5.77%, 29/503) (*P* < 0.0001). Also, ADV infection was detected more among children (74%, 37/50) compared to adults (22%, 11/50) (*P* > 0.05). Moreover, PV was detected more among children (86.67%, 26/30) compared to adults (10%, 3/30) and elderly (3.33%, 1/30), while it was completely absent among the adolescents (*P* > 0.05) (Table 3). In contrast, HSV1 was observed more among adults (50%, 6/12) compared to children (33.3%, 4/12) and adolescents (8.33%, 1/12) (*P* > 0.05) (Table 3). EBV infection was also detected more among adults (70.9%, 39/55) compared to children (18.18%, 10/55) and elderly (10.9%, 6/55) (*P* < 0.0001) (Table 3). Likewise, VZV infection was detected more among adults (51.42%, 18/35) compared to children (22.86%, 8/35) (*P* < 0.0001). Similarly, HSV2 was detected more among adults (85.71%, 12/14) compared to children (14.29%, 2/14) but not noticed among adolescents and elderly groups (*P* < 0.0001) (Table 3).

**Table 3:** Distribution of VM pathogens by age group.

	**Age Groups (years)**						
	**All groups**	** <1–9**	**10–17**	**18–59**	**>60**		
	**Positive cases**						
**Pathogen**	**No. (%)**	**No. (%)**	**No. (%)**	**No. (%)**	**No. (%)**	***X^**2**^***	*p*-value^*^
ADV	50 (100)	37 (74)	1 (2)	11 (22)	1 (2)	1.14	*P >* 0.05
HSV1	12 (100)	4 (33.33)	1 (8.33)	6 (50)	1 (8.33)	1.58	*P >* 0.05
HSV2	14 (100)	2 (14.29)	0 (0)	12 (85.71)	0 (0)	15.4	*P < * 0.0001
EBV	55 (100)	10 (18.18)	0 (0)	39 (70.9)	6 (10.9)	38.8	*P < * 0.0001
EV	503 (100)	428 (85.1)	45 (8.94)	29 (5.77)	1 (0.2)	111.7	*P < * 0.0001
CMV	33 (100)	22 (66.66)	0 (0)	11(33.33)	0 (0)	0.95	*P >* 0.05
VZV	35 (100)	8 (22.86)	2 (5.71)	18 (51.42)	7 (20)	36.7	*P < * 0.0001
PV	30 (100)	26 (86.67)	0 (0)	3 (10)	1(3.33)	1.07	*P >* 0.05
Total	732	537	49	129	17		

* *P-values were obtained using Extended Mantel-Haenszel chi-square test for linear trend*.

### Clinical Outcomes of Viral Agents

**Figure 4:**
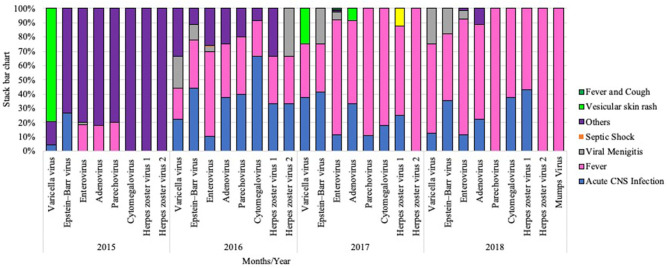
Stacked bar chart representing clinical manifestations among each VM cases during the study period from 2015 to 2018.

**Figure 5:**
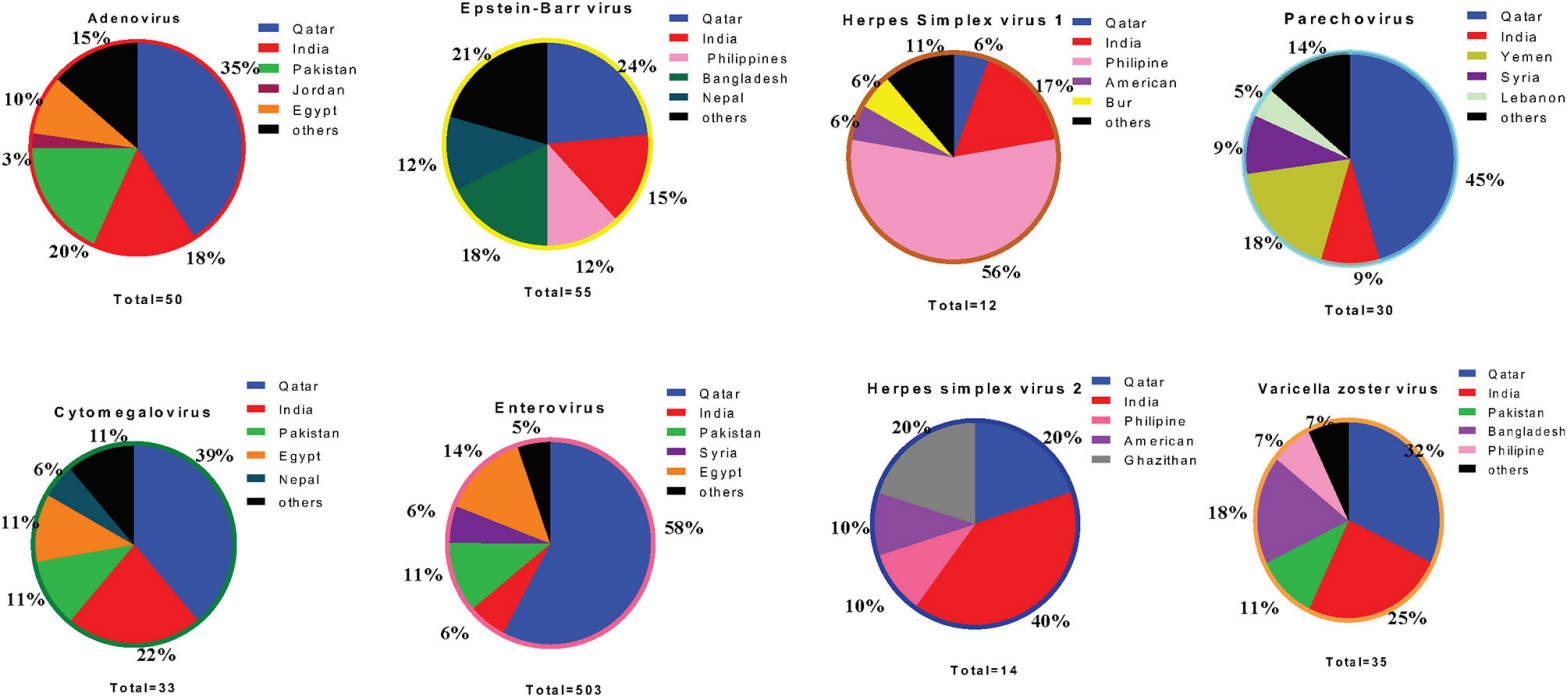
Pie chart representing percentage of top five population among each VM cases during the study period from 2015 to 2018.

Clinical outcomes of patients who were diagnosed with viral VM showed that fever (59.4%, 435/732) and acute CNS infection (15.6%, 114/732) were initial symptoms of most cases (Figure 4). VMLS (such as headache and stiff neck) were observed in 4.9% (36/732), and those with other non-specified symptoms (such as gastroenteritis, urinary tract infection, and others) were 19.5% (143/732). Rare symptoms such as vesicular skin rash and exanthematous skin rash were seen in 0.4% (3/732) and 0.27% (2/732) of infected subjects. Analysis of clinical manifestations revealed that meningitis-like symptoms, acute CNS infection, and other non-specified symptoms were detected among all patients regardless of causative VM agent, while VMLS was observed among patients positive for EBV (1.1%, 8/732), EV (3.1%, 23/732), VZV (0.5%, 4/732), and HSV2 (0.14%, 1/732). On the other hand, septic shock was noticed only in one patient positive for EV (0.14%, 1/732). Vesicular skin rash was noticed in two patients with VZV (0.3%, 2/732) and one patient with ADV infection. Fever and cough-like symptoms were observed only in two patients positive for EV (0.27%, 2/732) (Figure 5).

### Nationality Wise Distribution of Suspected VM

We ran an analysis to study the correlation between circulating VM viruses and the patients' nationality. Among the five major nationalities in this study, the most prevalent EV infection rates were higher among subjects from Qatar (39.1%), Egypt (9.4%), Pakistan (8.9%), India (8.6%), and Syria (3.7%) (Figure 5). Analysis of other VM pathogens revealed that ADV infections were more prevalent among individuals from Qatar (30%), followed by Pakistan (18%) and India (14%). EBV infections were detected at a higher frequency among the Qatari population (21.8%), Bangladesh (12.7%), and individuals from India (12.7%). Similarly, PV infection was observed at a high rate among the Qatari population (33.3%), Yemeni population (16.7%), and the Jordanian population (10%) (Figure 5). Besides the Qatari population, Indian nationality presented the highest infections with CMV (21.2%) and VZV infection (8.6%). Notably, HSV1 infection was significantly higher in Indian populations (25%) compared to both the Qatari population and the rest of the other nationalities. Likewise, Indian nationalities (35.7%) had a higher frequency of HSV2 infection, followed by the Qatari population (21.4%) and Ghanaian population (14.3%) (Figure 5).

## Discussion

VM is one of the most severe clinical conditions affecting people at various ages. In adults, VM is self-limited and is undetectable in most cases. However, in infants and children, developing of severe complications, such as high fever and mental retardation, is common and might lead to death in some cases. EV infections are responsible for most VM infections (90%), which is about one billion infections each year worldwide (19, 20). Regional information linking VM with patients' demographic data, underlying conditions, causative agents, etiology, and treatment susceptibility is essential for correct and timely management of this infection. In this study, we attempt to draw a comprehensive clinical picture of the situation of VM in Qatar.

The study spans a period of 4 years and is the first of its kind from the multi-national State of Qatar (80% of the population are expatriates). Presented data illustrates the epidemiological profile of major viral agents and their distribution among subjects suffering from VMLS. Out of the 6705 CSF samples collected from suspected patients during 2015–2019, 732 (10.9%) positive cases tested positive for a viral agent. Compared to our study, high rates of VM were reported in several countries in the MENA region, including Jordan (83%; 2001–2014) (21), Lebanon (82.7%; 2008–2016) (22), Gaza (72%; 2013–2014) (23), Egypt (43%; 2013–2015), United Arab Emirates (UAE) (37%; 2000–2005) (24), and Oman (31%; 2000–2005) (25). Apart from the MENA region, various Western countries have also reported high VM positive cases compared to our study. This includes the England and Wales study (2011–2014), which reported 36% positive VM cases (26), and a Canadian study (1998–2007), which reported 30% positive VM cases (27).

Regarding gender analysis, this study demonstrated higher VM infections among males (60.9%) compared to females (39.1%). A similar observation of higher incidence of VM among males was previously reported from MENA studies, including Oman (2000–2005) (25), Tehran (1999–2005) (28), Lebanon (2008–2016) (22), and Saudi Arabia (1994–1996) (29). Concerning age, children (<1–9 years old) were the most affected group accounting for 65.5% of all VM positive cases. Likewise, a study conducted in Tunisia showed that the average age of infected patients with VM was 6.1 years old (30). However, the majority of published studies in many countries, such as the US (31), UK (32), and Denmark studies (33), and from the neighboring countries, such as Kuwait (34) and Palestine (35), showed that infants <1 year of age are the most affected age group due to their immature immune systems.

Over the 4-years study period (2015–2019), 68.7% (503/732) of VM infection cases were mainly attributed to the EV. Similar epidemiology has been reported in several MENA countries such as Tunisia (63.4%) (30) and Kuwait (24%) (34). On the other hand, only Jordan showed a low prevalence of EV compared to countries of the MENA region (36). This discrepancy can be attributed to several factors, such as study population, sample type, method sensitivity, and type of data (sporadic cases or outbreaks of aseptic meningitis) (37). EV positive cases prevailed mainly among children (85.1%), followed by adolescents (8.94%) and adults (5.77%) (*P* < 0.0001). These results were consistent with the reports from Palestine (2012–2015) (35) and UAE (2000–2005) (24). In comparison, HSV2 and EBV infection significantly prevailed among adults with 85.71 and 70.9%, respectively.

*Following EV and EBV (7.5%), ADV (6.8%) indicated the highest dominance rate*. ***One*** *timely issue with ADV is the interference of pre-existing Ad5 with the immunogenicity of some COVID-19 vectored vaccines* (38). Other viral agents, including VZV, PV, HSV-1, and HSV-2, were detected but at lower frequency, about 4.8, 4.1, 1.6, and 1.9%, respectively. Studies have shown that the viral etiology of VM differs from one geographic area to another. For instance, HSV ranks second in adolescents and adults in developed countries like France, England, Spain, and the USA (9, 39–41), while EBV-type 1 is prevalent in Western Europe and EBV-type 2 is prevalent in central Africa, Papua New Guinea, and Alaska (42). On the other hand, VZV and PV are reported worldwide; however, population-based data observed a higher infection rate among high-income countries (43–45). These significant differences in incidence rates can be attributed to the different geographical conditions, age groups, type of surveillance system, time of the study, and immunization status prevailing in the countries for detecting VM cases (46).

Concerning VM seasonality, minimal studies were reported from the MENA region. In general, our data indicated that VM infection circulated throughout the year. Notably, we observed higher infection rates during the onset of spring seasons and a drop during winter seasons. Our findings are consistent with Johnson et al. who reported that VM cases' seasonal dynamics are highest during the summer and the early fall^5^. On the other hand, a recent study from Iran reported higher circulation rates in cold months (fall to spring) (46). The association between VM seasonality and climate conditions may not be well-evaluated, as many of the cases are not updated nor reported to public health authorities^5^. As mentioned previously, EV was the primary causative agent of VM in Qatar, and it was detected throughout the year and peaked significantly during spring seasons (March–June). Based on national enterovirus surveillance data collected by the US Centers for Disease Control and Prevention (CDC) during 1983–2013, EV circulates year-round but tends to peak in summer (47). Similarly, seasonal study reports from Palestine (2012–2015) (35), Saudi Arabia (1989–1995) (48), Tunisia (1992–2003) (49), and Lebanon (2009–2012) (50) presented slight seasonal pattern with more cases occurring during spring and summer, but still, significant numbers were also reported in fall and winter seasons. In this study, we could not observe any unique pattern for EBV circulation; likewise, the virus seasonality was less interpretable for the other VM agents with no clear temporal patterns of infection.

Analysis of VM infection patterns within the major 10 nationalities present in the study (Indians, Egyptians, Pakistanis, Syrians, Bangladeshis, Nepalese, Filipinos, Sudanese, Jordanian, and Sri Lankans) showed variable infection frequencies. Based on nationality, expatriates (60.9%) showed a higher number of VM cases than the Qatari population (39.1%). Besides Qatari population, Indian nationality presented highest infections rate with HSV2 (40%), CMV (22%), and VZV infection (25%). Notably, HSV1 infection was significantly higher in Indian populations (25%). Of note, the difference in infection rates probably reflects the distribution of these infections in patients' countries of origin (51, 52).

In summary, the current VM retrospective study provides comprehensive information concerning VM distribution in Qatar during 2015–2018. Our findings reflect, to a great extent, the incidence of viral meningitis infection in Qatar. These findings provide an excellent generalizability characteristic with direct implications to develop VM's efficient control and prevention programs before hosting the World Cup 2022. Moreover, this study surpasses other studies from the region regarding the mixed population involved, the association of demographic features, exploiting the seasonality distribution, and finally associating the clinical manifestations. Considering seasonality, Qatar's hot vivid climate seems to report a different trend of VM agent's circulation compared to other regions. Besides this, the spectrum of Qatar residents' multinational composition may also attribute to the circulation of certain viruses.

## Data Availability Statement

The raw data supporting the conclusions of this article will be made available by the authors, without undue reservation.

## Ethics Statement

This study was conducted in full accordance with the regulation of research at Hamad Medical Corporation (HMC) and Qatar University (QU): HMC-Institutional Review Board (HMC-IRB approval # 17288/17) and QU-IRB (QU-IRB 998-E/18). The ethics committee waived the requirement of written informed consent for participation.

## Author Contributions

HY, KA, AA, and MA developed the concept project. JN helped in retrieving the data from MOPH. SM, HA, NY, and HY wrote the initial draft of the manuscript. All authors revised the manuscript and approved it before submission for publication.

## Conflict of Interest

The authors declare that the research was conducted in the absence of any commercial or financial relationships that could be construed as a potential conflict of interest.

## Footnotes

^1^Aseptic Meningitis.

^2^Meningitis V. Medscape. (2018).

^3^Infectious Disease, antimicrobial agents.

^4^Openepi.

^5^RP J. Aseptic meningitis. UpToDate (2003).

## Supplementary Material

Supplementary Figure 1 Gender distribution of the identified viral agents associated with meningitis in Qatar between 2015–2018.

Supplementary Figure 2 Prevalence of circulating VM agents associated with age groups represented in pie chart. **(A)** Enterovirus, **(B)** Epstein-Barr virus, **(C)** Adenovirus, **(D)** Cytomegalovirus, **(E)** Herpes simplex virus type 1, **(F)** Herpes simplex virus type 2, **(G)** Parechovirus, **(H)** Varicella zoster virus.
