# Supplementary material for: Epidemiology Profile of Viral Meningitis Infections Among Patients in Qatar (2015–2018)
Source: Front Med (Lausanne). 2021 Jun 16;8:663694. doi: 10.3389/fmed.2021.663694 (PMC8241925; doi:10.3389/fmed.2021.663694)
Supplement: Supplementary Figure 1 — Gender distribution of the identified viral agents associated with meningitis in Qatar between 2015–2018. [file Presentation_1.pptx]

## Slide 1
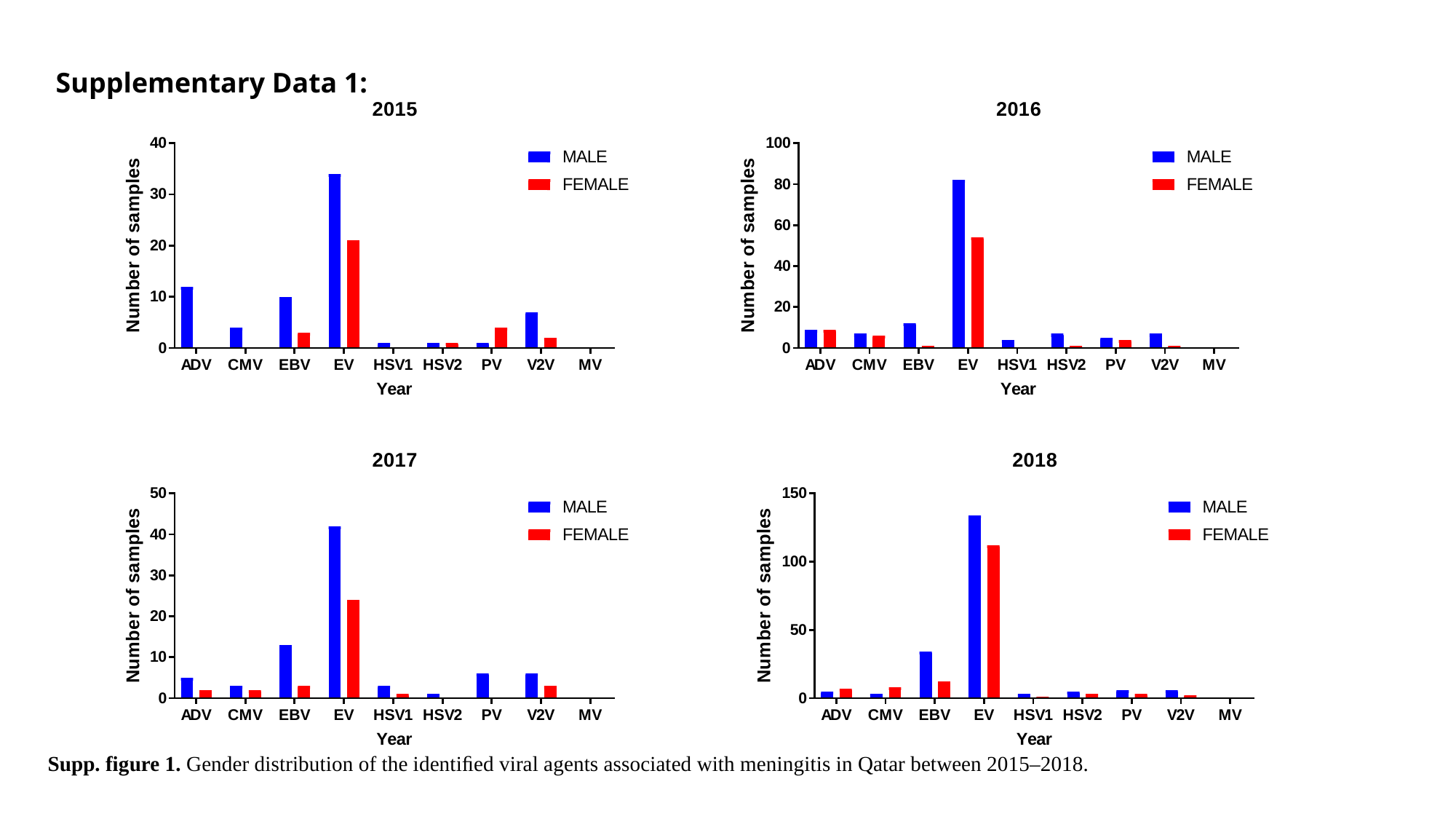

# Supplementary Data 1:
Supp. figure 1. Gender distribution of the identiﬁed viral agents associated with meningitis in Qatar between 2015–2018.

## Slide 2
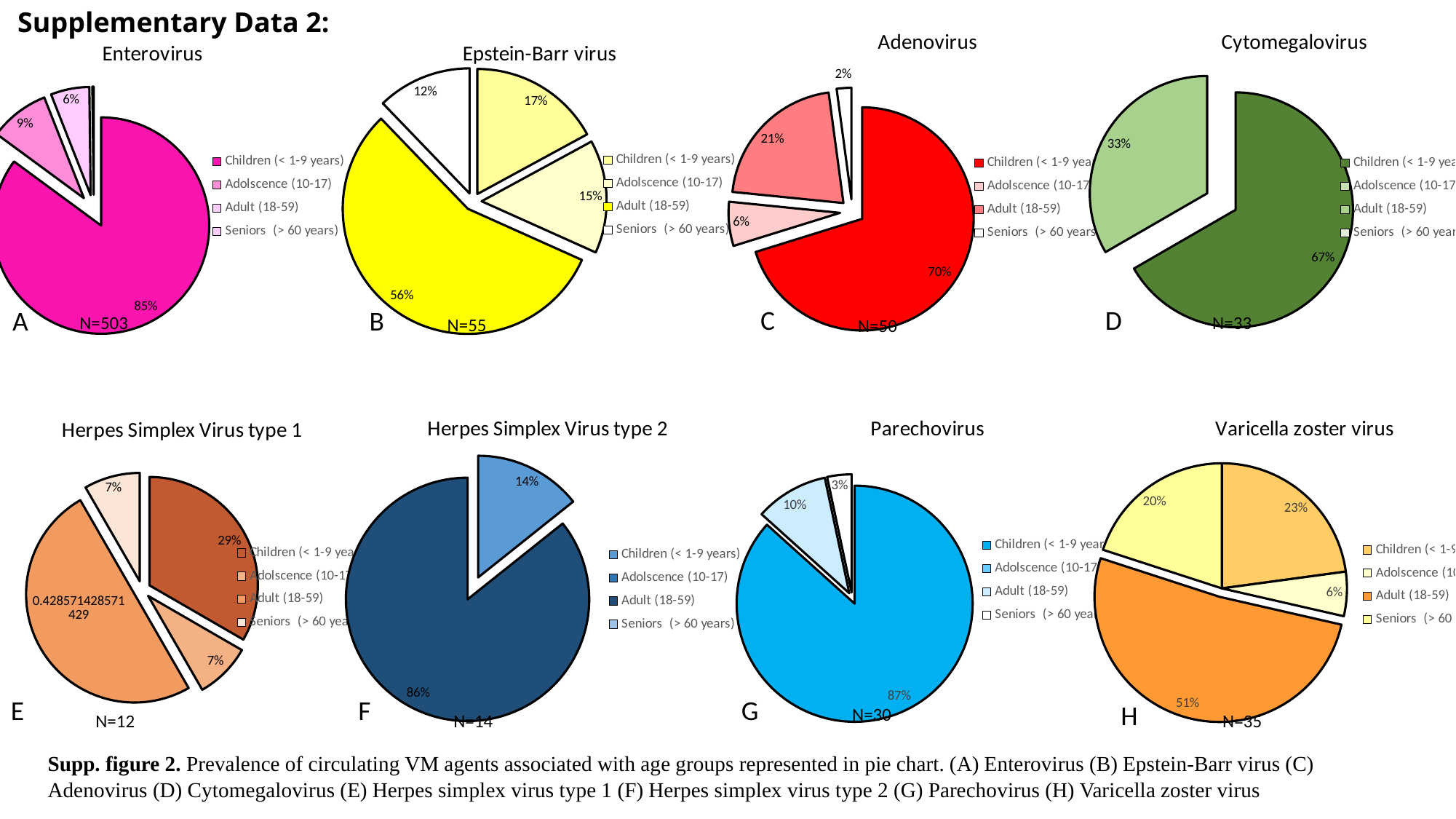

# Supplementary Data 2:
### Chart:
| Category | Enterovirus |
|---|---|
| Children (< 1-9 years) | 0.8508946322067594 |
| Adolscence (10-17) | 0.08946322067594434 |
| Adult (18-59) | 0.05765407554671968 |
| Seniors (> 60 years) | 0.0019880715705765406 |
### Chart:
| Category | Epstein-Barr virus |
|---|---|
| Children (< 1-9 years) | 0.17073170731707318 |
| Adolscence (10-17) | 0.14634146341463414 |
| Adult (18-59) | 0.5609756097560976 |
| Seniors (> 60 years) | 0.12195121951219512 |
### Chart:
| Category | Adenovirus | Adenovirus |
|---|---|---|
| Children (< 1-9 years) | 33.0 | 0.7021276595744681 |
| Adolscence (10-17) | 3.0 | 0.06382978723404255 |
| Adult (18-59) | 10.0 | 0.2127659574468085 |
| Seniors (> 60 years) | 1.0 | 0.02127659574468085 |
### Chart:
| Category | Cytomegalovirus |
|---|---|
| Children (< 1-9 years) | 0.7096774193548387 |
| Adolscence (10-17) | 0.0 |
| Adult (18-59) | 0.3548387096774194 |
| Seniors (> 60 years) | 0.0 |C
D
A
B
N=33
N=503
N=55
N=50
### Chart: Herpes Simplex Virus type 2
| Category | Herpes Simplex Virus type 2 |
|---|---|
| Children (< 1-9 years) | 0.14285714285714285 |
| Adolscence (10-17) | 0.0 |
| Adult (18-59) | 0.8571428571428571 |
| Seniors (> 60 years) | 0.0 |
### Chart:
| Category | Parechovirus |
|---|---|
| Children (< 1-9 years) | 0.8666666666666667 |
| Adolscence (10-17) | 0.0 |
| Adult (18-59) | 0.1 |
| Seniors (> 60 years) | 0.03333333333333333 |
### Chart:
| Category | Varicella zoster virus |
|---|---|
| Children (< 1-9 years) | 0.22857142857142856 |
| Adolscence (10-17) | 0.05714285714285714 |
| Adult (18-59) | 0.5142857142857142 |
| Seniors (> 60 years) | 0.2 |
### Chart:
| Category | Herpes Simplex Virus type 1 |
|---|---|
| Children (< 1-9 years) | 0.2857142857142857 |
| Adolscence (10-17) | 0.07142857142857142 |
| Adult (18-59) | 0.42857142857142855 |
| Seniors (> 60 years) | 0.07142857142857142 |E
F
G
H
N=30
N=35
N=14
N=12
Supp. figure 2. Prevalence of circulating VM agents associated with age groups represented in pie chart. (A) Enterovirus (B) Epstein-Barr virus (C) Adenovirus (D) Cytomegalovirus (E) Herpes simplex virus type 1 (F) Herpes simplex virus type 2 (G) Parechovirus (H) Varicella zoster virus
